# Atypical cells parameter in Sysmex UN automated urine analyzer: feedback from the field

**DOI:** 10.1186/s13000-021-01068-5

**Published:** 2021-01-22

**Authors:** Ozgur Aydin

**Affiliations:** Central Laboratory, Kepez Public Hospital, Hüsnü Karakaş Mahallesi, No:124, Güneş Cd, 07320 Kepez, Antalya, Turkey

**Keywords:** Atypical cells, Automated analyzer, Cancer, Cytology, Urinalysis, Urothelial carcinoma

## Abstract

**Background:**

“Atypical cells” parameter in automated urinalysis has recently been introduced. An instrument capable of measuring quantitative and qualitative features of nuclear and cytoplasmic properties of a cell has the potential to detect cellular atypia. Instruments using flow cytometry have been detecting atypical cells in blood for a long time; yet instruments using the same methodology very lately developed this parameter in urinalysis.

**Materials and methods:**

Samples with an atypical cells value higher than 1 atypical cell/µL were included in the study. Besides automated urinalysis, every sample was reflexed to modular unit for digital imaging. The remainder of each sample was stained with Sternheimer dye and examined manually under a light microscope.

**Results:**

50 samples with higher than1 atypical cell/µL result were included in the study. Patients were composed of 43 females (86 %) and 7 males (14 %). The mean age was 47.12 ± 19.45 years. The median atypical cells value was 1.8/µL (95 % range 1.5–2.4/µL). Manual microscopic evaluation of the 50 samples showed atypical cells in 1 sample. The patient had papillary lesions on cystoscopy and pathology report informed a high grade urothelial carcinoma. Other 49 samples were negative for atypical cells in manual microscopy. They were crowded samples with leucocytes and squamous epithelial cells.

**Conclusions:**

The positive case provided evidence for Sysmex UN’s capability to detect atypical cells in urine. The negative cases presented clues that probable vulvovaginal contamination and crowded specimens could be deceptive for Sysmex UN in this particular parameter.

## Introduction

It’s exciting to see urinalysis technology evolve [1]. Manual microscopy in urine sediment analysis is still respected as the gold standard but apparently straggle between automation and manual examination seems to be over in clear advantage of machines. Machines are fast, cost effective and efficient. It is not odd to define some machine work, particularly performed by automated urinalysis instruments as artificial intelligence. The software systems of these instruments are programmed to define particular cell types in urine as much alike the way human brain does. Some of them use optic lenses built inside to “see” while some others use laser beams and voltage sensors to compose a sense of “vision”. The vision is then evaluated (which is a clear act of intelligence) by software systems that label the cells to compose a report. Of concern, last generation devices dare to add clinical diagnosis estimations to their reports.

Bladder cancer is the eighth most common cancer in the World. The pathogenesis is complex and multifactorial [2]. The bladder, a store of waste products, accumulates harmful chemicals filtered from the bloodstream by the kidneys. External factors like smoking and prolonged exposure to aromatic amines are confirmed to lead neoplastic progression of the urinary epithelium. Microscopic examination of urine sample is commonly used to determine several conditions that can affect the urinary tract. The observation of fresh urinary sediments allows the identification of diverse cellular types associated with varied pathologies including carcinomas [3]. Automated instruments are designed to report a series of parameters like red blood cells, white blood cells and epithelial cells in routine of a laboratory. Epithelial cells are differentiated according to their origin in some instruments but “atypia” of these cells is not a common parameter to be reported.

Sysmex UN-Series automated urinalysis instruments challenge diagnosing neoplasms of urinary track. “Atypical cells” is a research parameter, namely reported by the instrument but not validated or presented in the patient reports. This study aims to participate to the efforts in evolution of the so-called research parameter that will hopefully enhance patient care.

## Materials and methods

Sysmex UN-Series (Sysmex Corporation, Kobe, Japan) present a modular system which allows configuration of instruments that best suits the workflow needs of laboratories. The reflexive and complementary combination of technology allows laboratories to harness the walkaway efficiency of automated particle counting via flow cytometry but still allows for reflexing to digital image review for those abnormal samples that require it. This study was run by a combination of Sysmex UC-3500, UF-4000 and UD-10. The UC-3500 uses test strips for chemical analysis of the urine. UF-4000/5000 uses fluorescence flow cytometry technology and hydrodynamic focusing for urine sediment analysis, where particles are stained by specific fluorochromes for nucleic acids and surface structures and then sent through the semi-conductor laser beam. Counting and classification is based on signals of scattered light and fluorescence to determine the characteristics of the particles. Atypical cells show side fluorescence and scattered light properties indicating their enlarged nuclei and increased nucleus/cytoplasm ratio. If more investigation of certain particles is requested UD-10 presents images captured by an internal camera.

Empirical threshold value of 1 atypical cell/µL by the UF-4000 analyzer was decided to define samples to be included in the study. The cut-off value was due to observations and results of a simple statistical study where all atypical cells results higher than 0.0/µL in 3 randomly selected consecutive days were evaluated. Accordingly, any sample with an atypical cell value higher than the threshold was reflexed to UD-10 unit for digital imaging. The remainder of the sample was centrifuged at 1500 rpm for 5 minutes, the pellet was stained with Sternheimer Urine Sediment Dye and then 20 µL was placed onto a microscope slide. Several low and high power fields were searched for atypical cells. UD-10 gave 40 high power field (HPF) images of a sample. Images from manual microscopy were also saved.

Every patient included in the study was searched for clinical findings, radiology, pathology and cystoscopy reports through the laboratory information system while manual microscopy was accepted to be the gold standard to define samples as positive or negative for atypical cells. Every patient included in the study was presented with the urinalysis test result, result of research parameters including the atypical cells, 40 HPF images of UD-10 and images of manual microscopy.

Data were analyzed using IBM SPSS 21 and Microsoft Excel 2013 to calculate results.

## Results

In order to decide on a cut-off value to select samples to include into the study: test results on Sysmex UN were searched in 3 consequent days. The instrument gave results of 627 samples sent to the laboratory from in-patient and out-patient clinics of the hospital for urinalysis in these 3 days. Of 627 patients, 85 patients (75 females, 10 males; mean age 40.77 ± 20.36 years) had “atypical cells” values greater than 0.0/µL (13.55 %). The median atypical cells value was 0.1/µL (95 % CI 0.1–0.3/µL) and the mean value was 0.46/µL (95 % CI 0.32–0.61/µL). 50 patients with an atypical cells value higher than 1/µL and enough residual sample volume for manual microscopic examination were evaluated as the subjects of this study. Patients were composed of 43 females (86 %) and 7 males (14 %). The mean age was 47.12 ± 19.45 years. The median atypical cells value was 1.8/µL (95 % range 1.5–2.4/µL).The percentage difference between genders were statistically significant in both (p < 0.0001).

**Fig. 1:**
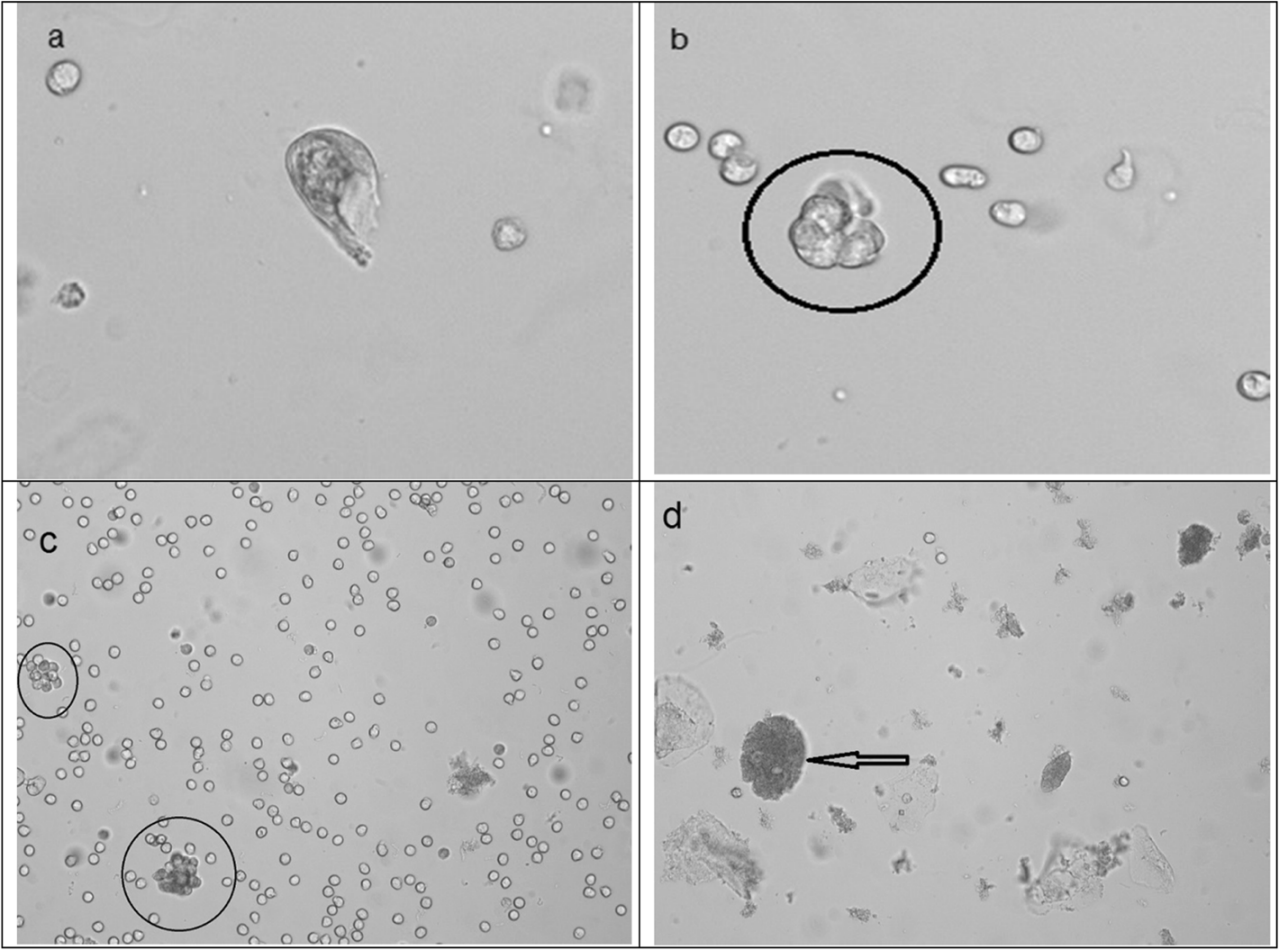
Original high power field digital images of Sysmex UD-10 unit are seen. A single atypical cell (**a**) and a group of atypical cells (**b**) belonged to the sample taken from the male patient with a high grade urothelial carcinoma. Sysmex UF-4000 flow cytometry unit gave 5.7 atypical cells/µL result for the patient. In (**c**) a crowded sample from a 26 years old female patient was seen. Almost all cells were white blood cells that formed a clump of cells as reported by the instrument. Her result was 5.5 atypical cells/µL. A 51 years old female patient showed a large number of squamous epithelial cells some of which were covered with bacteria (**d**). Her result was 13.7 atypical cells/µL. Samples of these two female patients were examined by digital images taken by UD-10 unit and by manual microscopy of stained slides. No atypical cells were detected

Manual microscopic evaluation of the 50 samples showed atypical cells in 1 sample. The patient was a 81 years old male. He had admitted to our urology outpatient clinic due to hematuria. Ultrasonography revealed a mass lesion suspicious for malignancy (rule out hematoma). Urinalysis showed chemical and cytological features of gross hematuria and urinary tract infection besides 5.7/µL atypical cells (Fig. 1). On cystoscopy examination, mucosal irregularities and papillary formations were observed. The lesions were excised and pathology report informed a high grade urothelial neoplasm.

Other 49 samples were examined in detail but were negative for atypical cells in manual microscopy. 40 HPF UD-10 pictures of each patient were reviewed and they were also negative for atypical cells.

## Discussion

Most of the urothelial neoplasms form papillary structures. Single or small groups of neoplastic cells can easily spill into the urine. However, atypical cells are very rarely reported in samples sent to the laboratory for urinalysis. It is not because these cells are absent in the samples but because they are not looked for. Unfortunately, finding and reporting atypical cells in urinalysis has not been a common concern for laboratory specialists, so that the literature is limited to a few case reports [3]. Manual microscopy has been replaced by full automated instruments for some time and manufacturers tend to improve their devices in line with the requests of laboratories. It is not surprising that manufacturers in the market have no interest in cancer diagnosis. Currently, Sysmex UN is the single instrument that presents the “atypical cells” parameter. A new instrument with a new research parameter means almost no literature.

Urine samples are subject to different approaches in two units of the central laboratories. Urine cytology examinations almost completely focus on cancer diagnosis [4] while urinalysis examinations almost completely ignore cancer diagnosis [3]. Urine cytology is quite different from urinalysis in sample preparation and evaluation. The cost effectiveness of the procedure is questioned and criticized for low performance [5]. Particularly considering urine cytology, using Sysmex UF in aid or even in replace of manual examination may be an option in the future [6]. Urinalysis testing requires fresh urine samples for chemical and microscopic examinations in which unstained sediment is used in general. The test mainly focus on number of red blood cells, white blood cells, epithelial cells per HPF and presence of bacteria, crystals, cylinders or some other particles. Unfortunately atypical cells have never been a common test parameter.

The laboratories host automated urine analyzers with an exuberant welcome. Apparently, human medical care service in laboratory is time-consuming, requires well-trained personnel and is subject to subjectivity and intra-observer variability. Automation in urinalysis has been relatively late in development and manufacturers face extra problems compared to other systems. Urine sediment analysis requires identification and decision steps which can very well be classified as artificial intelligence. Software systems evolve and new instruments dare to “suggest” clinical diagnoses or “warn” for clinical decisions. In daily practice, atypical cells are not requested as a parameter from an automated urine analyzer. However, analyzers like Sysmex UN share almost the same flow cytometry technology with automated hematology analyzers which report atypical cells for a long time. In peripheral blood or urine, flow cytometry is capable of measuring quantitative and qualitative features of nuclear and cytoplasmic properties of any cell [7].

In 2015 Anderlini et al. presented an inspiring study in which expert pathologists reviewed digital images of specimens in an automated urine analyzer [8]. The analyzer used a very similar flow cytometry technology to Sysmex UN but lacked the atypical cells parameter so that the experts had to review a bulk of images classified as “unclassified”. In a 5-year period, they reviewed 1,635,287 samples and reported atypical cells in 162 patients: representing an incidence of 0.1/1000 samples. The researchers also presented some clues in evaluating black and white images of the instrument. The present study was respectively lucky that a selective group of samples classified by Sysmex UN were reviewed. The cut-off value to define patients to be included in this study was empirical. Currently, there is no data to define a decision limit for this parameter. The single patient with a positive manual microscopy was confirmed by the post-operative pathology report as a high grade urothelial carcinoma. The case was very encouraging that true neoplastic cells can be detected by Sysmex UN. The other 49 cases were defined as negative due to manual microscopic examination as the gold standard. However, none of these patients underwent ultrasound examination or cystoscopy to confidently rule out neoplasm. These cases gave valuable information about features that were possibly deceptive for the instrument in this parameter. A striking feature of the patient group was the female predominance. 86 % of the subjects were female in this study. The percentage was in line with the pre-search study where 88 % of the patients with atypical cells results bigger than 0.0 /µL were female. Of concern, both rates contradict with the data that bladder cancer occurs 4.7-fold more frequently in men [2]. Apparently, samples of female patients owned some gender specific properties that were supposed to be deceptive in this parameter. Actually, urinalysis has some special considerations by gender because of anatomical differences. Contamination is annoying and mostly a feature of samples taken from female patients. While presence of squamous epithelial cells indicate contamination, there are no strict definitions or cut-offs to define a clear contamination in a sample [9]. Although the numbers were low to conclude statistical conclusions, only 1 in 7 samples from male patients contained squamous epithelial cells while the rate in female patients was 27 in 43. As an example, a 51 years old woman was presented with an atypical cells value of 13.7/µL (Fig. 1). Manual microscopy of her sample showed squamous cells some with clue cell morphology on a background of dense bacteria and leukocytes (31/HPF). The combination of bacteria, leukocytes and squamous epithelial cells could simply originate from vaginal discharges. Another feature shared by all samples was positive leukocytes. All 50 samples had leukocytes higher than the threshold (5/HPF). As explained in the instrument manual, signal pattern of atypical cells intermix with leukocytes forming groups. The software of the instrument defines particles according to signal waveforms created by scattered and fluorescence light that characterize the nuclear and cytoplasmic features. It is quite predictable that the signal pattern of a group of leukocytes can mimic the signal pattern of atypical cells. The same problem may also occur for clue cells which are squamous cells covered with bacteria.

In conclusion, atypical cells parameter of Sysmex UN deserves attention and care. This study should be perceived as a first look to this new item. Sysmex UN is a candidate to aid or replace urine cytology in the future [7]. However, the instrument serves for routine urinalysis in the laboratory and the ultimate goal should be implantation of the “atypical cells” parameter as a screening test. It should once again be emphasized that the instrument can and do detect atypical cells of urothelial carcinoma [10]. From a clinical point of view, a positive atypical cells result will have consequences like compulsory ultrasonography and/or cystoscopy examination of the urinary tract, besides the mental costs to the patient. The primary concern of future studies should be configuring a decision limit or a cut-off value. At this point the results of female patients should be evaluated with caution. Vulvovaginal contamination may be a potential risk for false positives which may be damaging for the reputation of an emerging parameter.

## Data Availability

Not applicable

## References

[1] Oyaert M, Delanghe J (2019). Progress in Automated Urinalysis. Ann Lab Med.

[2] Miyazaki J, Nishiyama H (2017). Epidemiology of urothelial carcinoma. Int J Urol.

[3] Fogazzi GB, Pallotti F, Garigali G (2015). Atypical/malignant urothelial cells in routine urinary sediment: worth knowing and reporting. Clin Chim Acta.

[4] Glass R, Rosen L, Chau K, Sheikh-Fayyaz S, Farmer P, Coutsouvelis C (2018). Analysis of the Cytomorphological Features in Atypical Urine Specimens following Application of The Paris System for Reporting Urinary Cytology. Acta Cytol.

[5] Tan WS, Sarpong R, Khetrapal P (2019). Does urinary cytology have a role in haematuria investigations?. BJU Int.

[6] Ren C, Wang X, Yang C, Li S, Liu S, Cao H (2020). Investigation of Atyp.C using UF-5000 flow cytometer in patients with a suspected diagnosis of urothelial carcinoma: a single-center study. Diagn Pathol.

[7] Aydin O (2020). Atypical cells parameter in an automated urine analyzer: Does it have a future?. Anal Biochem.

[8] Anderlini R, Manieri G, Lucchi C, Raisi o, Soliera AR, Torricelli F (2015). Automated urinalysis with expert review for incidental identification of atypical urothelial cells: An anticipated bladder carcinoma diagnosis. Clin Chim Acta.

[9] Mohr NM, Harland KK, Crabb V, Mutnick R, Baumgartner D, Spinosi S (2016). Urinary squamous epithelial cells do not accurately predict urine culture contamination, but may predict urinalysis performance in predicting bacteriuria. Acad Emerg Med.

[10] Aydin O, Yapıcı O, Copuroglu R. Atypical cells in sysmex UN automated.urine particle analyzer: a case report and pitfalls for future studies. Turk J Biochem 2020;45(5):617–19.

